# Designing Novel Strategies for Improving Old Legumes: An Overview from Common Vetch

**DOI:** 10.3390/plants12061275

**Published:** 2023-03-10

**Authors:** Elena Ramírez-Parra, Lucía De la Rosa

**Affiliations:** 1 Centro de Biotecnología y Genómica de Plantas, (CBGP, UPM-INIA/CSIC) Instituto Nacional de Investigación y Tecnología Agraria y Alimentaria, Consejo Superior de Investigaciones Científicas, Universidad Politécnica de Madrid, Campus de Montegancedo, Pozuelo de Alarcón, 28223 Madrid, Spain; 2 Centro de Recursos Fitogenéticos, (CRF-INIA/CSIC) Instituto Nacional de Investigación y Tecnología Agraria y Alimentaria, Consejo Superior de Investigaciones Científicas, Alcalá de Henares, 28805 Madrid, Spain

**Keywords:** *Vicia sativa*, common vetch, breeding, legume, genetic resources

## Abstract

Common vetch (*Vicia sativa* L.) is a grain legume used in animal feeding, rich in protein content, fatty acid, and mineral composition that makes for a very adequate component to enrich feedstuff. In addition, relevant pharmacological properties have been reported in humans. The common vetch, similar to other legumes, can fix atmospheric nitrogen, a crucial feature for sustainable agricultural systems. These properties enhance the use of vetch as a cover crop and its sowing in intercropping systems. Moreover, several studies have recently pointed out the potential of vetch in the phytoremediation of contaminated soils. These characteristics make vetch a relevant crop, which different potential improvements target. Varieties with different yields, flowering times, shattering resistance, nutritional composition, rhizobacteria associations, drought tolerance, nitrogen fixation capacity, and other agronomic-relevant traits have been identified when different vetch accessions are compared. Recently, the analysis of genomic and transcriptomic data has allowed the development of different molecular markers to be used for assisted breeding purposes, promoting crop improvement. Here, we review the potential of using the variability of *V. sativa* genetic resources and new biotechnological and molecular tools for selecting varieties with improved traits to be used in sustainable agriculture systems.

## 1. Introduction

**Figure 1:**
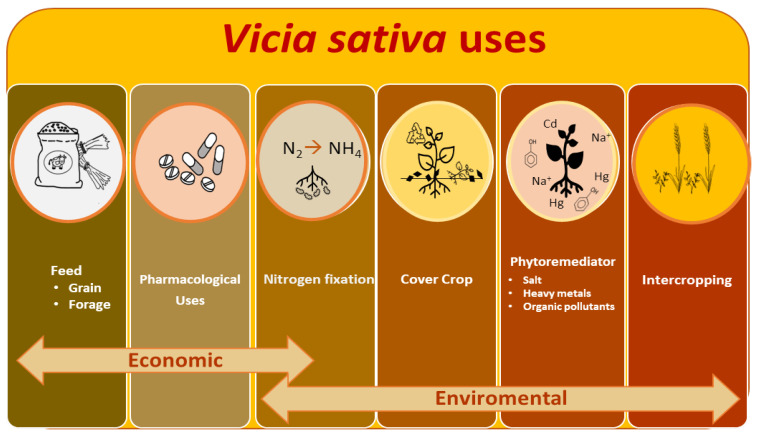
*V. sativa* main uses. The scheme includes economically relevant nutritional and medical uses. Environmental applications for sustainable agriculture systems are also incorporated.

The common vetch *(Vicia sativa* L., Tribe *Viciae,* Family *Fabaceae*) is one of the world’s most economically important annual grain legumes, used as animal feed, as forage (grain, hay, and for silage production) or as grain legume as a cheap and rich source of protein and minerals of high digestibility and high energy content [1,2]. Additionally, vetch fixes atmospheric nitrogen through its symbiotic interactions with rhizobia soil bacteria, which could improve the soil fertility significantly, making its cultivation appropriate in sustainable agriculture, as a main crop, as an element of rainfed rotation, as a cover crop, or in rotation with cereals [3] by decreasing the use of fertilizers and reducing CO_2_ emissions and other pollutants [4]. The main bottleneck of this species is the presence of antinutritional factors in various parts of the plant, but the enormous dependence on proteins of vegetable origin for animal feed and the interest in the EU for the use of species of environmental value make the use of this crop a relevant agricultural option with an essential role in the implementation of environmental measures such as From Farm to Fork Strategy and the new Common Agricultural Policy (CAP) directive. In this work, we have tried to review the status and evolution of vetch cultivation and gene bank genetic resources throughout the world from a historical, economic, and environmental perspective. We have reviewed the main nutritional and pharmacological uses and environmental benefits of this crop as a nitrogen-fixer legume in cover crops, intercropping, or soil phytoremediators (Figure 1). The main abiotic and biotic threats to this crop have also been explored. Finally, we present the status of the main genomic and transcriptomic tools, from a biotechnological perspective and by means of the use of plant genetic resources collections to address the challenge of vetch crop improvement through combined strategies.

## 2. Taxonomy

The botanical tribe Viceae, from the subfamily *Papilioboideae*, of the family *Fabaceae,* includes some genus of agricultural interest such as *Lens*, *Cicer, Pisum, Lathyrus*, and *Vicia* [5]. The genus *Vicia*, whose center of origin and diversification has been placed in the Mediterranean and Irano-Turanian regions [6], includes a number of species ranged between 150 [7] and 210 [8], from which 34 are cultivated. The genus is, nowadays, distributed in temperate regions of the northern hemisphere in Asia, Europe, and North America, and also in the non-tropical region South America [9]. This genus has an enormous phenotypic variation [10]; in fact, there has been a big number of taxonomic revisions made over the genus, more than 20 since the original classification presented by Linneo (1735–1770) in the 18th century, following for those of Jaaska and other authors [9,11,12,13,14,15]. In a focus on *V. sativa* section *Vicia*, which includes the most important agricultural species, one of the last classifications was proposed by Van der Wouw et al. [16] after several studies focus on *Vicia L*. series *Vicia*, who presented a classification of this series in four species: *V. babazitae* Ten&Guss., *V. incisa* M.Bieb., *V. pyrenaica* Pourr., and *V. sativa*, which includes six subspecies: *nigra* (L.) Ehrh., *segetalis* (Thuill) Čelak., *amphicarpa* L.Batt, *macrocarpa* (Moris) Arcang., *cordata* (Wulfen ex Hoppe) Batt., and *sativa.* This group of forms is named the *V. sativa* aggregate.

Commercially vetch includes, in addition to *V. sativa, V. villosa* Roth. (winter vetch, hairy vetch) and other species of similar local importance such as *V. pannonica* L. in Turkey, *V. pannonica*, *V. ervilia* L. and *V. articulata* Hormen in Spain, and *V. benghalensis* L. in Australia; the same term, vetch, has been used for different species such as *Lathyrus sativus* L. and other *Lathyrus* species in Africa [17]. In this paper, we will use the name vetch to refer exclusively to cultivated species included in the *Vicia sativa* aggregate.

## 3. A Historical Crop

The common vetch, similar to other species of the *Fabaceae* family, has been cultivated together with cereals since the beginning of agriculture. Archaeological evidences indicate the Mediterranean Basis as the center of origin and primary diversification of this species [18,19]. We present below an historical revision of vetch use based on the Spanish Inventory of Traditional Knowledge about Agricultural Biodiversity [20], one of the most complete reviews of vetch uses.

Some authors indicate that the first archaeological references to vetch seeds date back to the Neolithic Periodic and the Bronze Age. This point is not clearly established because these seeds could also belong to wild species, associated with the crops. Additionally, others authors such as Zohary [21] disagree with this approach, dating the use of vetch into the agricultural systems in the Roman Empire, at a time when the use of vetch as a fodder species as already been reported, together with others species such as alfalfa and lupin or fenugreek, also associated with cereals and others grain legumes [22]. Columela, an ancient Roman scientist and writer who lived in the first century B.C. cited the use of vetch for poultry (hens and pigeons) feeding and as a fodder and green manure, together with other legumes such as alfalfa and fenugreek [23]. In the same time period, Plinius The Elder (First century B.C.) said that their use would improve soil fertility, giving indications about the sowing times in the function to the final use, including the use as fallow [24]. This author mentioned that vetch was the best feed for the bullocks. In the 4th century, Paladio described the use of a mixture of lupin and vetch as a soil improver when cutting in green, and they also made mention of the differences in the sowing date of the function of the final use. Isidore of Seville, who lived between the 6th–7th century, highlighted the scarce production of seed of vetch compared with other legumes [25]. In the Middle Age (11th and 12th century), vetch was a minority crop in Europe [26], even if the Andalusian author Abü l-Jayr indicated names such as *Umda* or *Amank* to identify different forms of vetches [27].

In the 16th century, Juan de Járava wrote that this species could be found among cereals and that it could be eaten as lentils, although it did not taste good [28]. In this century, vetch traveled to the New World, adapting perfectly to the local conditions of America, to the point that some escapees from cultivated vetches came to grow wild in the new environmental conditions [26]. Thus, in the 19th century, vetch was introduced to Argentina by Italian immigrants (settlers) establishing it as a well-known fodder [29]. To conclude this historical revision, a book from the 18th century used several names for cultivated and wild vetches and mentioned that they were a well-known crop in Europe, and that they could have reached Spain from the east by crossing France. Here, also, its uses as grain, green manure, and as a flour component to make bread in times of scarcity are mentioned [30].

## 4. Worldwide Vetch Cultivation

**Figure 2:**
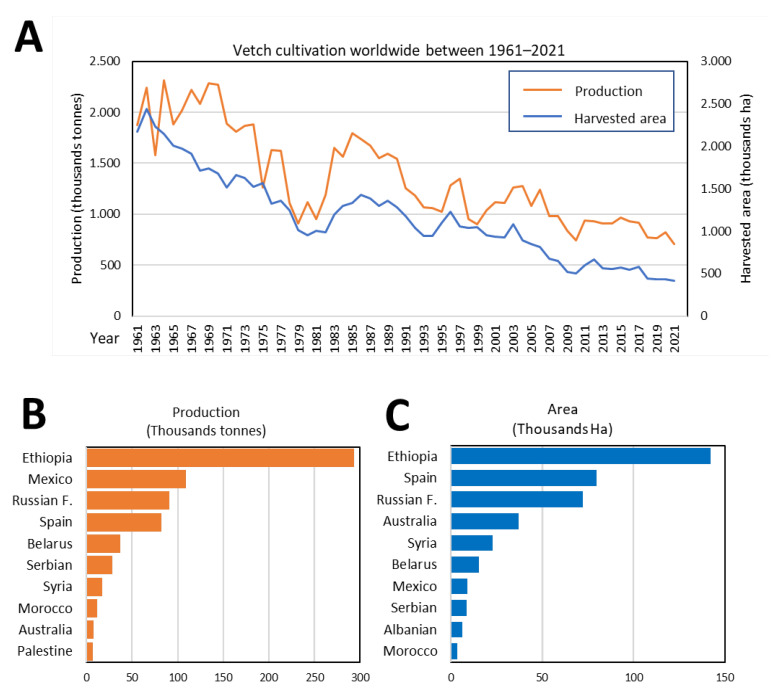
Worldwide *V. sativa* cultivation. (**A**) Production data and cultivated area of vetch worldwide during the last 60 years. Main producers according to cultivated area (**B**) or production (**C**) during the year 2021.

Due to its economic and ecological advantages (Figure 1), vetch is now widespread throughout many parts of the world. Figure 2A shows, based on data by FAOSTAT and the Spanish Ministry of Agriculture, Fisheries and Food [31], the surface and production of this crop from 1961 to 2021 are shown in Figure 2A. In the agricultural season of 2020–2021, the main producers were Ethiopia, the Russian Federation, Spain, Mexico, and Australia (Figure 2B,C).

According to FAO data from FAOSTAT, the economic value of the agricultural gross production of vetches worldwide was USD 139,237,000. This value was clearly well below the economic value of other legumes, partly due to its low production. Comparing its production worldwide with that of other legumes (average of last 5 years, 2017–2021), vetch production was 8 times less than lentil production or 18 times less than the production of chickpeas [31,32]. One of the reasons for this reduced production was the presence of antinutritional factors (ANFs) present in the grains. Therefore, as we discuss below, this is one of the main aims for common vetch improvement.

## 5. Nutritional and Pharmacological Properties

The nutritional value of the common vetch as a livestock feedstuff has been analyzed in different studies that have recently been reviewed [2,32]. The main conclusions of different works agree with the potential of common vetch grain, despite of the well-known deficit in sulfur amino acids (methionine and cysteine), as a rich source of proteins, minerals, and other nutrients, while being cheaper than other alternatives. The average crude protein values range from 21 to 39% (dry matter) and crude fat ranges from 9% to 38%, with high levels of palmitic and linoleic acids. The main essential and non-essential amino acids are leucine and glutamic acid, respectively. The seeds have high caloric content and are highly digestible [2]. These characteristics make vetch a potential nutrient-rich resource to be incorporated into animal diets and are very suitable to replace soy or a large proportion of cereals in certain feeds, maintaining their energy content. The nutritional content of the vetch seeds has been analyzed, and great differences in protein content, fatty acid composition, and mineral composition, including iron, were observed between accessions from different geographical origins. Although these studies have been carried out on a small scale, these data support the use of the variability of genetic resources from the gene banks of *V. sativa* for breeding purposes [33]. Remarkably, the large variation in crude protein and mineral content between different cultivars is much greater even than that due to climatic conditions [2,34,35]. This fact must be considered when selecting varieties with better nutritional conditions.

The medical uses of *V. sativa* have been also explored [36]. The seed flour and plant extract are traditionally used as an anti-poison and antiseptic [37,38], as an anti-asthmatic and respiratory stimulant in bronchitis [39], and as rheumatism treatment and an antipyretic [40]. Anti-acne [41] and antibacterial activity has been also validated [42]. However, most of the phytopharmacological mechanisms of action remain to be unraveled.

As described in other grain legumes, common vetch seeds contain a variety of antinutritional factors (ANFs), such as vicine, convicine, tannins, phenolic compounds, trypsin inhibitors, and cyano-alanines. Although some of these elements, such as polyphenols, have been studied as a source of antioxidants [43], these ANFs have partially limited the use of the seeds in food and/or feedstuffs, especially in the diets of monogastric animals [44]. However, the inclusion of a high proportion of common vetch seeds in the diet of ruminants does not produce relevant negative effects on their health [45,46,47,48,49,50]. The levels of anti-nutritional factors such as tannins, trypsin inhibitors, and hydrogen cyanide nutrients show huge variations between different accessions conserved in gene banks [33]. These variations have permitted the selection of low vicianine levels in common vetch accessions and have allowed the production of cultivars such as *Blanchefluer* without vicianine [51,52], extensively growing in Australia as a substitute for red lentils, although its consumption in humans is residual [17]. Last year, the molecular bases that regulate the hydrogen cyanide (HCN) synthesis from these cyanogenic glycosides have been unraveled in common vetch. Transcriptomic assays at different seed developmental stages enlighten important information about the regulatory network of this pathway. Eighteen key regulatory genes that are involved in HCN biosynthesis have been identified. These genes would be crucial as molecular markers for the selection and breeding of low HCN levelled vetch germplasm [53]. In any case, and especially for non-ruminant diets, it seems that these ANFs present in common vetch seeds need to be reduced or partially inactivated by adequate grain processing methods. A practical approach would be the selective breeding of varieties with a lower content of these antinutrients, but also the processing by soaking, chemical treatment, dehulling heat treatment, or germination. These treatments not only reduce the ANF content, but also improve the digestibility, palatability, and availability of the nutrients [35,54,55].

## 6. Environmental Benefits

The multiple benefits of common vetch for the farm as a versatile crop have been reviewed [2,32]. Plants need relatively large amounts of nitrogen for proper growth and development. The largest input of N into the terrestrial environment occurs through the process of biological nitrogen fixation (BNF). Therefore, BNF has great agricultural and ecological relevance, since N is often a limiting nutrient in many ecosystems [56]. The reduction of synthetic nitrogen fertilizers through the use of legumes not only has a decrease in environmental impact but also an economic one, due to the prices of these fertilizers, whose synthesis involves a large energy cost [57].

**Figure 3:**
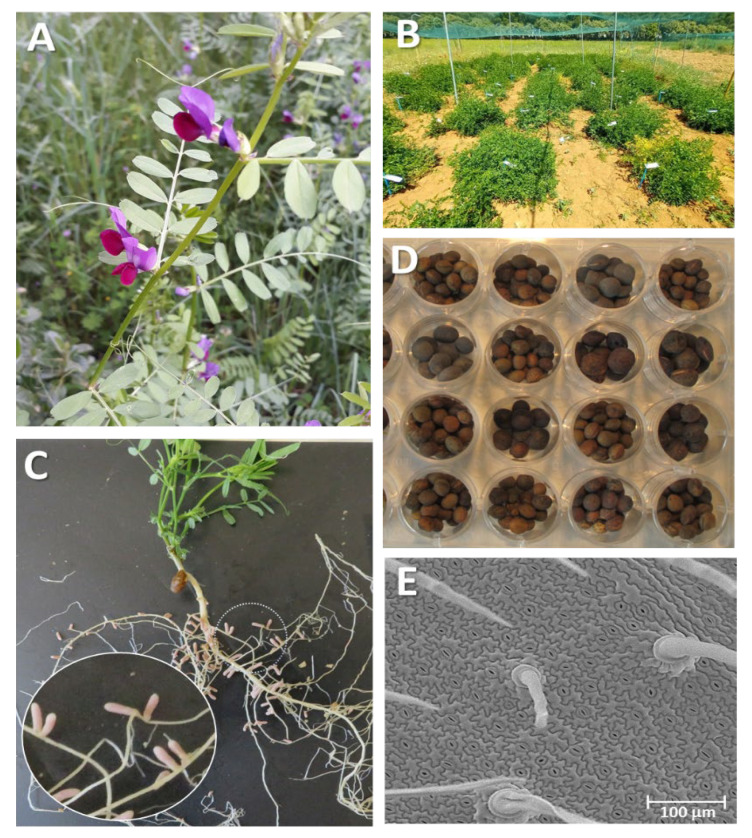
*V. sativa* plants showing different tissues and growing stages. (**A**) Wild-growing common vetch at “Sierra Norte”-Madrid (Spain). (**B**) Field evaluation assay of different accessions (CRF-INIA/CSIC gene bank). (**C**) Indeterminate *Rhizobium* nodules of a common vetch root. (**D**) Diversity of size, shape, and color observed in seeds from different accessions from a CRF core collection. (**E**) Abaxial leaf surface, showing trichomes, stomas, and epidermal cells.

Rhizobia from legume-symbiotic systems make use of its nitrogenase enzyme to catalyze the conversion of atmospheric nitrogen (N_2_) to ammonia (NH_3_), which is a plant assimilable nitrogenous compound. This process utilizes energy produced by the legume photosynthesis and takes place in the symbiotic nodules of the legume roots. As other species of the *Vicia* genus, common vetch forms indeterminate-type root nodules through symbiosis with rhizobia to promote nitrogen fixation (Figure 3C). The interaction between the bacteria and host legume is so intricate that many rhizobial species nodulate in a host-specific manner despite the fact that the same symbiotic bacteria can infect different species, and even different genera, of legume. *Rhizobium leguminosarum* biovar *viciae* (Rlv) is the most common symbiont of *V. sativa* in which effective nitrogen fixation has been validated [56]. Furthermore, different strains of the *Mesorhizobium* and *Bradyrhizobium* genus have been isolated from *V. sativa* nodules, although there are no data about their ability to fix nitrogen [58]. Specific rhizobial nodule establishment in the plant host not only depends on the strain abundance in soil but also their nodulation competitiveness. *R. leguminosarum* biovar *viciae* establishes symbiosis with several legume genera, and genomics studies reveal plant preferences between specific rhizobial genotypes and the host *V. sativa* [59]. The complexity of these symbiotic associations and their specificity have been extensively addressed. These interactions present differences between *V. sativa* cultivars and wild relatives and are also affected by environmental conditions [58]. Moreover, the analysis of symbiotic genes of *R. leguminosarum* isolated from *V. sativa* from different geographical locations reveals a common phylogenetic origin, suggesting a close coevolution among symbiotic genes and legume host in this *Rhizobium-Vicia* symbiosis [60]. Symbiosis within *V. sativa* and *Rlv* has also been chosen as a model system to analyze different bacterial compounds, mainly oligosaccharides, and the plant-produced *nod* gene inducers (NodD protein activating compounds) involved in the establishment of the effective symbiosis with its host plant and the requirements for the host-plant specificity [61,62,63,64]. The bacterial nodulation genes (*nod*) are activated by flavonoids excreted by the common vetch roots [65], and, subsequently, the plant responds with the development of the root nodule [66]. Several physiological, biochemical, and transcriptomic analyses support an increase in drought tolerance in nodulated vetch plants compared to non-nodulated ones. Transcriptomic analysis has helped to discover specific drought pathways that are specifically activated in nodulated *V. sativa* plants, improving the understanding of the impact of the symbiosis-associated genetic pathways on the plant abiotic stress response [67].

Crop systems in which legumes intercropped with cereals have traditionally been used in preference to legume or cereal monocultures, as it will result in higher forage yields and minimize synthetic fertilizers due to the nitrogen fixation ability of the legumes. The intercropping system of spring wheat (*Triticum aestivum* L.) with common vetch had a significant advantage on grain yield, beneficial effects on root development on both crops, and less N and P fertilizer requirements [68]. The use of vetch in the rotation of maize (*Zea mays* L.) and wheat helped to reduce the N deficiency, the increase in N concentration in the soil during next growing season, and the reduction in N losses by leaching [69]. Systems of oats (*Avena sativa* L.) or ryegrass (*Lolium multiflorum* Lam.) intercropped with common vetch have also proven to be especially profitable on dairy farms in central Mexico for silage cow feeding [70]. Finger millet *(Eleusine coracana L.)* is a widely grown cereal crop in some arid and semiarid areas in Africa, such as Ethiopia. Field assays in which ringer millet was intercropped with three vetch species, including *V. sativa,* concluded a general improving of the total dry matter yield and the quality of the intercrops [71,72,73]. Although the most well-characterized intercropped systems are those of legume cereal, other systems have been documented as intercropping with rapeseed (*Brassica napus* L.), which require higher levels of N fertilizers [73]. Additionally, the use of *V. sativa* in kiwifruit orchards increases the microbial community, moisture, and nutrients in the soil, activating plant growth [74].

Cover crops play an essential role in agroecosystems. They are unharvested plants grown in the gap between crops or integrated into rotations, which improve soil health, reduce erosion, enhance water availability, promote nutrient capture, are useful for controlling pests, weeds, and other diseases, and promote additional benefits for agriculture [75,76,77]. The use of legumes such as *V. sativa* as a cover crop allows the fixation of atmospheric nitrogen in the rhizobia symbiosis nodules, then the plant residue decomposes and remains available in the soil for the next harvest, acting as green manure by reducing the amount of inorganic fertilizer and reducing CO_2_ emissions [77,78]. It has recently been observed that *V. sativa* helps prevent water losses and soil erosion in vineyards (*Vitis vinifera* L.) [79]. In the USA, *V. sativa* is the most widely used legume cover crop [76]. In Argentina, *V. sativa* and *V. villosa* are the most important cultivated cover crop [80]. In Central Spain, the use of vetch as a cover crop in maize planted in the summer and autumn ensured the good production of principal crops and significant biomasses and N contents in the next following spring [81].

In recent years, environmental problems derived from soil and water contamination have begun to gain importance. In this context, the role that cover crops can have in phytoremediation is of great relevance [82]. *V. sativa*, together with *V. faba*, is the species of *Vicia* genus most frequently used in phytoremediation studies against inorganic and organic pollutants [83]. Different studies of phytoremediation, tolerance, and accumulation of inorganic and organic pollutants on *V. sativa* are summarized in Table 1. The relevance of *V. sativa* for the remediation of saline soils has recently been revealed. The phytodesalination process implies a high capacity of the plant to tolerate, absorb, and accumulate sodium in harvestable tissues [84]. Regarding the detoxification of organic compounds on *V. sativa*, the effect of the herbicide sulfosulfuron was evaluated without relevant effects on root or shoot growth parameters [85]. Similar studies assessed the effect of phenol and mepiquat chloride on seed yield and yield components in *V. sativa* plants, without drastic damages [86,87,88]. The growth, nodulation nitrogen fixation activity and *V. sativa* were less negatively affected by high concentrations of phenolics than in other tested legumes [89]. Wider studies on phytoremediation of diesel-fuel-contaminated soil were also developed in common vetch. The assays showed a greater tolerance of the vetch in diesel-contaminated soils and a greater capacity for decontamination of the soils compared to other crops [90]. Although *V. sativa* cannot be considered a hyperaccumulating plant capable of storing high concentrations of metals, copper tolerance has been described for germinative seeds [91,92]. Molecular mechanisms responsible for this tolerance remain to be explored, although it has been shown that vetch may prevent oxidative damage in the presence of some pollutants such as phenol by increasing the activity of lipid kinase and phosphatidic acid avoiding its toxicity [86,93]. *V. sativa* plants can also accumulate and concentrate different heavy metals. Curiously, these plants accumulate mercury (Hg) in the roots [94] but concentrate cadmium (Cd), lead (Pb), zinc (Zn), and nickel (Ni) in the aerial parts [95,96,97,98]. The tolerance of *V. sativa* to Cd seems to be related to antioxidant enzymes [99]. Phytochelatin synthases (PCS) and γ-Glutamylcysteine synthetase (γ-ECS) are directly involved in metal detoxification in plants. Ectopic overexpression of *V. sativa* PCS (*VsPCS1*) and γ-ECS (*Vsγ-ECS*) genes, which are Cd-inducible genes, are capable of increasing the tolerance to cadmium and the triggering of the detoxification pathway in Arabidopsis [100,101]. These results support the potential biotechnological use of these plants in phytoremediation processes against metal contamination.

**Table 1:** Summary of studies of phytoremediation, tolerance, and accumulation of inorganic and organic pollutants on *V. sativa*.

Pollutant	Developed Assay	References
Cd	Cd tolerance. Oxidative damage accumulation.	[96]
Cd and Zn	Zn and Cd accumulation in different tissues	[95]
Zn	Zn tolerance	[98]
Cu	Cu tolerance	[91,92]
Salt	Tolerance to salt. Na and K accumulation	[84]
Hg	Hg accumulation in different tissues	[94,100]
Ni	Ni accumulation. Oxidative damage accumulation.	[97]
Sulfosulfuron herbicide	Tolerance to sulfosulfuron	[85]
Diesel fuel	Tolerance to diesel	[90]
Phenol derivatives	Polychlorinated biphenyl (PCB) dissipation	[87]
Phenolics	Tolerance to phenolics. Effects on biomass, nodulation and nitrogen fixation activity	[89]
Mepiquat	Tolerance to mepiquat	[88]

It has recently been shown that the rhizosphere microorganisms associated with the vetch roots can synergistically increase the decontamination potential by maximizing the efficiency of the process [102,103,104]. However, it is necessary to analyze the possible synergies or antagonisms derived from symbiosis to improve their efficiency in phytoremediation. On some occasions, bacterial strains tolerant to different pollutants do not show this activity when they are in symbiosis with *V. sativa* [83].

## 7. Pests and Diseases on Common Vetches

Diseases cause losses in quality and yield of common vetch crops and include viral, bacterial, and fungal infections, and, also, insect, spider, and nematode pests [105].

Many of the insects of forage legumes attack vetches, including beetle, flies, and aphids, promoting direct injuries or causing indirect damages by being vectors of virus transmission [106]. The herbivorous beetle vetch weevil (*Bruchus rufipes* Herbst.) is an important pest for legumes, including vetches. Their larvae feed on the grains, reducing the germination capacity of the seeds. The beetle *Sitona lineatus* L. is also a *V. sativa* common pest. Adults promote leaf damage, and the larvae produce symbiotic nodule destruction at a root level. *Bemisia tabaci* (Gennadius) flies cause damage to the crop, due to the suction of sap and the injecting of toxins through their saliva, which causes an overall weakening of the plant. *Delia platura* (Meigen), the seedcorn maggot, or the seed fly, is a polyphagous that significantly attacks vetch. Their larvae feed on seeds, young shoots, seedling stems, and roots, causing a general weakening or even vetch death. The pea moth (*Laspeyresia nigricana* Fabricius) is a lepidopteran that also attacks vetch. Their larvae cause damage to the pod and grain, promoting premature yellowing and the loss of its germinative power. Aphids also attack vetches. Thus, the black legume aphid (*Aphis craccivora* Koch) and the green legume aphid (*Acyrthosiphon pisum* Harris) attack young shoots, biting and sucking the sap, causing leaf deformation, shoots yellowing, and reducing photosynthesis. Other arthropods, such as the red spider mite (*Tetranychus urticae* Koch.), also promote severe damage to *V. sativa*. The affected leaves decrease their photosynthetic and transpiration capacity, causing defoliation, especially at the first phases of the crop that are the most sensitive period to the attack of this spider [107].

Fungal infections are the diseases most likely to cause the greatest losses in vetch. An extensive review has been carried out on fungal diseases affecting vetch, such as anthracnose, powdery mildew, rust, and botrytis [105]. At least 14 fungal diseases on *V. sativa* had been reported from 28 countries [108]. Over 43 fungal pathogenic species infect common vetch. These pathogens belong to the Deuteromycotina (58%), Ascomycotina (16%), Basidiomyotina (14%), and Mastigomycotina (12%) groups. Anthracnose is the main fungal disease in common vetch and is mainly caused by six pathogenic fungi: *Colletotrichum vicia* Dearness, *C. villosum* Weimer, *C. sativum* Horn, *C. vicia-sativa* Sawada, *C. lentis* Damm, and *C. spinaciae* Ellis & Halst. [108].

In vetch, there are no known severe diseases caused by bacterial pathogens. Bacterial blight was one of the first and more relevant infections identified in *V. sativa*. The disease, recorded in Australia for the first time, was caused by *Pseudomonas stizolobii* Wolf [109] that promotes necrotic lesions on stems, leaves, and flowers, which, in many cases, causes the complete wilting of the plant [110].

No severe economic losses associated with viral infections in common vetch crops have been reported. However, different viruses capable of infecting *V. sativa* and promoting effects on the quality and quantity of production have been identified [111]. *Artichoke Yellow Ringspot Virus* (AYRSV) infection in *V. sativa* was reported from Greece and Italy. This virus (*Nepovirus* genus; *Secoviridiae* Family) promotes severe stunting and leaf mottling in the infected vetch plants [112]. Additionally, *Bean Yellow Mosaic Virus* (BYMV) infections have been identified in Germany a long time ago. BYMV (*Potyvirus* genus; *Potyviridae* Family) promotes several symptoms, including leaf and stalk necrosis, distortion of plants, and dark to yellow green stripes along the veins on the lower surface. The virus is transmitted by aphid vectors in a non-persistent manner [113]. *Broad Bean Stain Virus* (BBSV) infection was reported from Slovakia in *V. sativa* crops. BBSV (*Comovirus* genus; *Secoviridae* Family) is transmitted by weevils [114]. *Chickpea Chlorotic Stunt Virus* (CpCSV) infection has been identified in *V. sativa* crops from Syria. Symptoms in virus-infected vetches include exhibit yellowing, reddening, and stunting. This virus (*Polerovirus* Genus; *Luteoviridae* Family) is transmitted by aphid vectors in a circulative, non-propagative manner [115]. For the *Faba Bean Necrotic Yellows Virus* (FBNYV), *V. sativa*-infection was identified from Azerbaijan. The virus-infected vetch plants exhibit leaf rolling, yellowing, and stunting symptoms. FBNYV (*Nanovirus* Genus; *Nanoviridae* Family) is transmitted by aphid vectors in a persistent but non-propagative manner [116].

Annual and parasitic weeds are the most important constraints for legume production, including vetches, because of their slow initial growth that causes poor competition to weeds. In the case of parasitic weeds, broomrapes (*Orobanche* and *Phelipanche spp.)* are obligate parasites that infect roots of dicotyledoneous plants, including *Vicia* genus. Broomrapes represent severe weed problems causing relevant yield losses on crops. *Orobanche* spp. are particularly important in Southern and Eastern Europe, the Middle East, and North Africa. *Orobanche foetida* Viv. is considered important as an agricultural parasite of common vetch crops in the western Mediterranean area, including Portugal, Spain, Morocco, Algeria, and Tunisia [117]. Recently, *O. crenata* Forssk. has been identified as a parasite on vetch crops in Spain [118]. Different components isolated from *V. sativa* root exudates were reported as stimulants of *Orobanche* or *Phelipanche sp.* seed germination [119]. The basis of host resistance to broomrapes is almost unknown, but vetch resistance based on inducing necrosis of broomrape tubercles by the formation of mucilage and the occlusion of host xylem has been reported [120]. The dodders (*Cuscuta* spp.) are also damaging parasitic plants of vetch crops that are highly susceptible to *C. campestris.* However, recently, some resistant genotypes have been identified in *V. sativa* germplasm collections [121].

The use of pre-emergence herbicides is effective, but the use in post-emergence is scarce due to their lack of safety [122] and the shortage of available active substances [123,124]. An additional constraint in some regions is the strict limitation on the use of plant protection for weed control (Council Directive 2009/128/EC of the European Parliament and of the Council of 21 October 2009 that establishes a framework for Community action to achieve the sustainable use of pesticides). Strengthening research is the most adequate way to improve this situation, both in the search of environmentally friendly active ingredients as well as in the design of varieties with the genetic capacity to compete with weeds.

## 8. Germplasm Gene Banks and Common Vetch Genetic Diversity

Gene banks are relevant resources for the conservation of natural genetic diversity and provide a source of novel features for fluctuating circumstances associated with climate change and new disease outbreaks and are crucial for sustained crop improvement [125,126,127].

**Figure 4:**
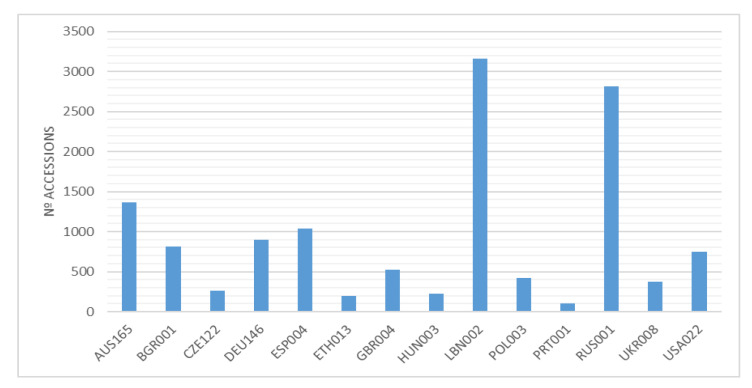
Main collection of *Vicia sativa* accessions, by holding institution, reported at GENESYS. The holding institutions are identified by their WIEWS code, available at https://www.fao.org/wiews/data/organizations/en; (accessed on 15 February 2023).

The total number of varieties and accessions of common vetch ex situ conserved is quite difficult to estimate due to the taxonomic complexity of this species. GENESYS (https://www.genesys-pgr.org/ accessed on 15 February 2023) is an online platform that includes information on Plant Genetic Resources (PGR) for food and agriculture conserved in gene banks worldwide, and it was accessed in February 2023. This data base includes a total of 54,743 accessions that belong to the genus *Vicia*, 13,694 of which belong to the species sativa, including the next taxons: *V. sativa*, *V. sativa* subsp *sativa*, *V. sativa* subsp *nigra*, *V. sativa* subsp *cordata*, *V. sativa* subsp *amphicarpa*, *V. sativa* subsp *macrocarpa, V, sativa* subsp *incisa*, *V. cordata*, *V angustifolia*, and *V. macrocarpa,* most of them landraces and wild forms. Regarding the provenance, these accessions has been mainly collected in Russia, Turkey, Spain, Italy, Syria, and Bulgaria. Figure 4 shows the number of accessions by holding institutions.

Regarding Europe, EURISCO, the European catalog of plant genetic resources (https://eurisco.ipk-gatersleben.de/ accessed on 15 February 2023), includes a total of 7943 accessions named *V. sativa*, including also the species and some subspecies. The most important collections are those kept at the gene banks of the Russian Federation, Germany, Spain, and Bulgaria.

Despite the abundance of genetic resources, the situation regarding the availability of commercial varieties of vetch, at least in Europe, is limited. The European Common Catalogue (https://ec.europa.eu/food/plant-variety-portal/ accessed on 15 February 2023), includes 110 commercial varieties and 2 conservation varieties of this crop, registered by 14 countries. Italy, France, and Spain are the countries with the highest number of registered varieties.

One of the limiting factors for the use of these germplasm collections is the lack of characterization data; another limitation is the possible environmental influence on the expression of the agro-morphological traits. The solution to increase the value of these resources is the genotyping of these collections and the use of molecular marker-assisted selection.

## 9. Generating Genomic and Transcriptomic Tools

Recent years have witnessed the development of different types of increasingly efficient molecular markers that have allowed the characterization of the diversity of the accessions present in collections around the world (Summarized in Table 2). The first works were based on the use of retrotransposon-derived Sequence-Specific Amplified Polymorphism (SSAP) markers and allowed a preliminary characterization of the genetic diversity of the genus *Vicia* [128]. The use of Amplified Fragment Length Polymorphism (AFLP) derived markers allowed the diversity analysis of the genetic singularity coefficients in Russian *V. sativa* varieties [129]. Seed reserve protein patterns were also used to characterize the diversity present in the germplasm on a collection of Spanish vetches [130]. Start Codon Targeted (SCoT) markers have been used for analyzing the intra-population diversity of several common vetch varieties and optimizing the minimal sample size to assess their genetic diversity [131]. Inter Simple Sequence Repeats (ISSR) markers have been also used for characterizing more than 25 accessions from the Spanish *V. sativa* germplasm collection [132]. During the last few years, the rapid emergence and efficient development of next-generation sequencing (NGS) technologies have allowed the transcriptomic analysis of many species in a high-throughput manner with reasonable economic costs and in a time-efficient approach. This methodology has made the in-depth analysis of gene expression and the annotation of a large number of genes possible, but it has also been very useful for analyzing the presence of polymorphisms, managing to design molecular markers as simple sequence repeats (SSRs) and single nucleotide polymorphisms (SNPs) associated with functional transcribed genes and associated traits [133,134]. Thus, the analysis of *V. sativa* transcripts from RNA-seq data has allowed the identification of cDNA-SSR molecular markers with a high degree of polymorphisms [135,136,137]. These markers have shown a high amplification potential in other species of the *Vicia* genus [138], supporting the possibility of using heterologous markers for diversity analysis of related species. These SSRs are mapped in coding regions of the genome, less polymorphic but potentially more conserved. This fact could favor the high rate of transferability across closely related species of the same genus. Recent works have developed a minimum set of 14 SSR reference alleles that have allowed the genotyping of over 545 common vetch worldwide accessions, including landraces, cultivars, and wild relatives. This analysis has allowed an exhaustive analysis of the diversity present in this collection based on which a Spanish vetch core collection (Figure 3B,D) has been created with a minimum loss of genetic diversity in comparison with the total collection [132]. Genome-derived polymorphisms have also been identified. Over 24,000 single nucleotide polymorphisms (SNP) have been identified from 1243 plants of the 12 natural *V. sativa* Japanese populations, and double-digest restriction-site associated DNA sequencing (ddRAD-Seq) was used to evaluate heterogeneity of these accessions [139]. A total of 76,810 different SSR have been also identified, tri- (36%) and tetra-nucleotide (13%) repetitions being the most abundant. Some of these SSR have been used to analyze the genetic diversity of Chinese common vetches [140].

**Table 2:** Summary of molecular markers used for diversity characterization of *V. sativa* germplasm.

Type of Molecular Marker	Target	References
Retrotranspon-derived Sequence-Specific Amplified Polymorphism (SSAP)	Genomic sequences	[128]
Amplified Fragment Length Polymorphism (AFLP)	Genomic sequences	[129]
Seed reserve protein patterns	Protein	[130,132]
Start Codon Targeted (SCoT) marker	cDNA sequences	[131]
Inter Simple Sequence Repeats (ISSR)	Genomic sequences	[132]
cDNA-SSR	cDNA sequences	[132,135,136,138,140,141,142]
SNP	cDNA sequences	[132,139,142]
double-digest restriction-site associated DNA sequencing (ddRAD-Seq)	Genomic sequences	[139]
genomic-SSR	Genomic sequences	[140]

### 9.1. Genomic Data and Transcriptomic Characterization of Some Traits and Developmental Stages

The lack of a high-quality and complete publicly available reference genome sequence of *V. sativa* has restricted the advances in molecular breeding and functional genomics efforts to improve this crop. Previous publications have reported controversial karyotypes with at least three different chromosome numbers (2n = 10, 12, and 14) inside the *V. sativa* aggregate. The four main taxa, *sativa, macrocarpa, cordata*, and *angustifolia*, suggest taxonomic ascription problems into the *V. sativa* aggregate or spontaneous amphidiploids or hybrid derivatives [143,144].

During this last year, two groups have sequenced the common vetch genome. Recently, a draft of common vetch chromosome-level genome has been published with a partial 85% assembly of over 1.5 Gb and 31,146 predicted genes [139], validating a ploidy of 2n = 14 for the sequenced cultivar (*V. sativa subsp. sativa Cv. Studenica*). Another lab has reported similar results with an estimated sequenced genome size of 1.6 Gb [140]. Further efforts will make it possible to have a complete and annotated genome, which will make it easier to have efficient genomic tools. Meanwhile, different transcriptional approaches by RNA-seq have been developed, making it possible to have gene expression data and sequences of transcripts from different tissues, developmental stages, and stress responses. These results have been very useful to understanding at genetic level processes such as flowering time, floral development, pod shattering, metabolic processes associated with HCN synthesis, or plant responses to different abiotic stress as drought, salt, or cold, and their interconnection between different plant tissues as root and leaves, summarized in Table 3, whose results are explained below.

Flowering time is an important determinant of harvesting time. In common vetch, early flowering promotes plant seed production, but it affects the yield of forage biomass. Therefore, understanding the flowering process is crucial for breeding purposes. To unravel the molecular mechanisms of flowering regulation, *V. sativa* accessions with different flowering times were analyzed at different developmental stages for integrative analyses of the transcriptomes and metabolomes. Among the differentially expressed genes (DEGs), synthesis and signal transduction of plant hormone pathways were the most enriched pathways. Moreover, the contents of three metabolites related to salicylic acid biosynthesis correlated with the observed differences in DEGs [145]. The development of the zygomorphic flower of *V. sativa* (Figure 3A) has also been analyzed at the transcriptional level by comparative expression analyses on six floral organs (sepals, dorsal petals, lateral petals, ventral petals, stamens, and carpels) in common vetch. Results show that these gene expression patterns of the vetch flower fitted a strict ABC model, similar to the core eudicots *Arabidopsis* [146].

The seed dispersion by pod shattering is the main form of propagation of many wild species and is one of the plant characters radically changed by crop domestication, already studied in legumes such as soya [147], chickpea [148], and common bean [149]. This behavior is frequent in cultivated *V. sativa*, and it is one of the most important defects that limits its utilization [150]. To better understand the pod shattering mechanism at a molecular level, comparative transcriptomic analysis of pod ventral sutures between shattering-susceptible and shattering-resistant vetch accessions has been performed. The most enriched pathways among the differentially expressed genes were processes related to cell wall modifications and hydrolases associated with pod shattering. The results helped to unravel the pod-shattering gene regulation networks in vetch. This information is relevant for the identification of pod-shattering-related genes and the design of future molecular markers assisted selection in breeding programs [142,151].

### 9.2. Genomic Data and Transcriptomic Characterization of Stress Responses

The physiological response of *V. sativa* germplasm collections has been explored under treatments with different concentrations of NaCl to select salt-tolerant accessions for breeding programs. Salt-tolerant vetches have a higher K^+^/Na^+^ ratio than salt-sensitive plants under these treatments. To unravel molecular mechanisms involved in salt tolerance, the expression of genes involved in ion homeostasis was evaluated. Salt-tolerant varieties had higher expression levels in the ion transporters NHX7, HKT1, AKT2, and HAK17, compared with the salt-sensitive ones. Proline levels, expression of the enzyme P5CS1 involved in proline biosynthesis, and antioxidant enzymes SOD, CAT, and APX were also higher in salt-tolerant varieties [152]. A comparative transcriptomic analysis of the leaves and roots of common vetch under salinity stress was also performed. This assay has allowed us to begin to unravel the complex molecular mechanisms associated with the salt stress response and the differences and interconnections that are established between the aerial and radicular parts of the plant and how these can help explain the behavior observed in some salt-tolerant varieties [153].

Drought is one of the main stresses that threatens current agriculture. It is estimated that its effects will be more dramatic in the coming years, as a direct consequence of climate change. Drought pressures vetch production, both in forage and grain. Therefore, it is important to understand the mechanisms of response and tolerance to drought in this legume. Physiological drought responses of *V. sativa* have been analyzed at a photosynthetic [154,155] and biochemical level [142]. Recent years have witnessed numerous studies addressing the drought response from a molecular perspective, trying to understand the genetic networks involved. Transcriptomes carried out on whole tolerant and sensitive vetch plants under different drought treatments reveal a functional enrichment of genes involved in relevant process as “oxidation reduction”, “lipid metabolism”, “oxidoreductase activity”, or “plant hormone signaling” [142]. Additionally, the aquaporin gene family seems to play an essential role during drought stress [156]. Moreover, miRNAs that are small noncoding RNAs that negatively regulate the expression of downstream target genes have been recently identified as regulators of the drought response in *V. sativa*. Potential targets of the identified drought-responsive miRNA include genes involved in various pathways, as cell wall biosynthesis, reactive oxygen removal, and protein transport, providing new insights into the miRNA-mediated regulatory networks of drought stress response in common vetch [155,157]. The response to drought stress is complex and involves different gene regulatory networks and physiological responses in the root and the aerial parts that are responsible for water uptake and stomatal evapotranspiration, respectively (Figure 3E). Comparative transcriptomic analyses have been performed in common vetch leaves and roots under drought stress. These studies shed light on the coordinated response of aerial and radicular tissues of common vetch to drought stress. Hormone signal transduction, starch and sucrose metabolism, and arginine and proline metabolism were extensively enriched in genes belonging to drought-responsive pathways, including the enzyme P5CS1, involved in the biosynthesis of proline. Various TFs’ (Transcription factors) family members (WRKY, bHLH, AREB/ABF, MYB, and AP2/ERF) seem to be crucial regulators in the crosstalk between leaves and roots during drought response. Previous studies support that the expression profile in the roots was more stable than that in the leaves. However, both the aerial and root parts coordinate the gene response to optimize the whole-plant adaption in drought stress by undergoing similar biological processes [155]. Similar approaches were performed combining drought and cold abiotic stresses, analyzing the crosstalk between aboveground and underground parts of common vetch. This study identifies specialized and unique responses to combined stresses in common vetch [158]. To understand the molecular networks involved not only in drought response, but also in adaptive mechanisms of drought tolerance, transcriptomic differences and specific polymorphic variants (mainly SNPs and SSRs) between tolerant and sensitive accessions under drought have been analyzed. This strategy has allowed the design of drought-associated markers to be used as new molecular breeding tools [142].

**Table 3:** Developmental, metabolic, or stress processes with transcriptomic analysis in *Vicia sativa*. Analysis was performed at indicated plant tissue or developmental stage.

Process	Analyzed Plant Tissue	References
Flower Development	Floral organs (dorsal, lateral and ventral petals, sepals, stamens, carpels) leaf, and roots	[146]
Flowering time	Aerial part at different stages	[145]
Pod Shattering	Pod ventral sutures	[151]
Drought Tolerance	Whole plant under different drought treatments	[137]
Drought Stress	Root, stem, and leaf tissue under PEG treatments	[156]
Drought Stress	Comparative leaf versus root	[155]
Drought response and tolerance	Aerial part of tolerant and sensitive varieties	[142]
Cold–drought combined stress	Comparative leaf versus root	[158]
Drought Stress	Aerial part under PEG treatments	[157]
Salinity Stress	Leaf versus root	[153]
Hydrogen Cyanide Synthesis	Seed development	[53]

## 10. Perspectives for Future Breeding Strategies

Leguminous crops face several challenges, in the context of achieving sustainable agriculture. Among the main ones, it included the improvement of different agronomic and nutritional traits in common vetch by using several approaches. Traditional strategies have included the selection of cultivars with high forage and/or grain production. Additionally, the selection of varieties with flowering times or pod shattering adapted to different climatic conditions. Nowadays, to cope with increasing climate change in arid areas, breeding strategies for more tolerant and resilient crops, including common vetch, will be essential. The different tolerance mechanisms and management of drought stress and other associated abiotic stresses in grain legumes have been thoroughly reviewed and analyzed [159]. Legume crops face the challenge of managing water deficits by creating different ways for efficient water management at the same time as increasing crop yield [160]. Selecting drought-tolerant genotypes is strategically crucial to cope with water deficits. Breeding approaches as a result of the combination of traditional–classic techniques with novel tools of breeding techniques would generate yield improvements under drought. The use of classical breeding tools includes the exploitation of the diversity of plant genetic resources that is present in gene banks, including the use of crop wild relatives, for screening and selecting improved accessions. Novel strategies involve the use of biotechnological and molecular tools. *V. sativa* is particularly recalcitrant to transformation, thus obtaining transgenic plants transformed with *Agrobacterium tumefaciens* has been extremely difficult. Therefore, up to now the improvement in *Vicia* has been only reported by conventional methods. Hopeful results have only recently been achieved through the development of systems based on the use of *R. rhizogenes* for in vitro hairy root transformation by infecting different explants [161]. New approaches to cope with drought in legumes include the construction of transgenic legumes or Genome Editing (GE) tools, and also include the novel molecular and genomic techniques of Genome-Wide Association Studies (GWASs), Marker-Assisted Selection (MAS) with molecular markers, Genomic Selection (GS), and OMICs-based technologies by using transcriptome, genome, phenotype, proteome, and metabolome data as biotechnological tools. Within this strategy, several works have reported information on different drought-associated molecular markers (mainly SSRs and SNPs) in *V. sativa* to be used as new breeding tools for the molecularly assisted selection. Analysis of agronomic traits associated with drought have been carried out over a *V. sativa* core collection representative of the genetic diversity of a 545 whole collection (Figure 3B), selecting promising accessions.

In addition to these combined breeding strategies, new agronomy approaches are available. These tools include the exogenous application of plant Growth Regulators (PGRs), osmoprotectants, and bioinoculants of Plant-Growth-Promoting Rhizobacteria (PGPR). The improvement of biological nitrogen fixation by rhizobium symbiosis and the selection of new generation of highly effective rhizobia inoculants are some of the main challenges of sustainable agriculture research. The importance of the symbiotic interaction between legumes and rhizobium has promoted the production of commercial rhizobia inoculants. Inoculation of leguminous crops with rhizobia strains not only promotes sustainable farming systems by reducing fertilizer inputs, but also a considerable increase in crop yields from an economic and environmental point of view. In addition to selecting legume genotypes especially tolerant to abiotic stresses, another focus of the study is that the potential selection of rhizobia more tolerant to these types of stresses. This symbiotic systems with selected strains of *Rhizobium* could increase the legume production under drought conditions [67,162]. Selecting varieties with a high capacity for nitrogen fixation would be of interest to sustainable agriculture. In this sense, common vetch has shown novel applications and great potential for soil improvement in recent years linked with its ability as a phytoremediator, capable of reducing organic and inorganic pollutants in the soil, mediated by their roots’ bacterial communities. This capacity makes vetch a promising crop for improving soil health by decreasing contaminants.

Another potential target of improvement, as previously discussed, is associated with selecting varieties with increased nutritional properties. The large variations observed in protein and mineral content between different varieties open a promising gateway for selecting varieties with these characteristics. We must not forget that these selection strategies must correlate with the reduction in anti-nutritional factors, including tannins, trypsin inhibitors, and hydrogen cyanide, which also present variability between different varieties. Together with this, some authors have reported the use of *V. sativa* seeds as a source of functional components, mainly polyphenols, for the elaboration of functional foods [163]. The use of classical procedures to reduce the ANFs content (heating, washing, germination, fermentation, soaking…) and the new ones such as the Controlled Pressure Drop, ultrasound, or microwaves that could be used as an alternative to the cooking and germination to reduce the level of ANF [164] could be used to increase the use of legumes in general, and vetch, in particular, for animal and human consumption.

## Data Availability

Publicly available datasets were analyzed in this study. This data can be found here: https://www.fao.org/faostat/en/(accessed on 15 February 2023), https://www.genesys-pgr.org/ (accessed on 15 February 2023) and https://eurisco.ipk-gatersleben.de/apex/eurisco_ws/r/eurisco/home; (accessed on 15 February 2023).

## Abbreviations

AFLP: Amplified Fragment Length Polymorphism; ANF: Antinutritional Factors; AYRSV: Artichoke Yellow Ringspot Virus; BNF: Biological Nitrogen Fixation; BYMV: Bean Yellow Mosaic Virus; CAP: Common Agricultural Policy; CpCSV: Chickpea Chlorotic Stunt Virus; ddRAD-Seq: double-digest Restriction-site Associated DNA sequencing; DEG: differentially expressed genes; FAO: Food and Agriculture Organization; FBNYV: Faba Bean Necrotic Yellows Virus; GE: Genome Editing; GS: Genomic Selection; GWAS: Genome-Wide Association Studies; HCN: hydrogen cyanide; ISSR: Inter Simple Sequence Repeats; MAS: Marker-Assisted Selection; NGS: Next-Generation Sequencing; PCS: Phytochelatin synthases; PGPR: Plant-Growth-Promoting Rhizobacteria; PGR: Plant Genetic Resources; SCoT Start Codon Targeted; SNP: Single Nucleotide Polymorphisms; SSAP: Sequence-Specific Amplified Polymorphism; SSR: Simple Sequence Repeats; TF: Transcription Factors; γ-ECS: γ-Glutamylcysteine synthetase.
